# Supplementary material for: Limited column formation in the embryonic growth plate implies divergent growth mechanisms during pre- and postnatal bone development
Source: eLife. 2024 Sep 13;13:e95289. doi: 10.7554/eLife.95289 (PMC11509684; doi:10.7554/eLife.95289)
Supplement: Supplementary file 1. [file elife-95289-supp1.docx]

**Supplementary File 1. Mean and std for bone elongation and expansion measurements.** Distance from the longitudinal origin was used to represent bone elongation and equivalent radius was used to represent bone expansion. P; proximal, D; distal, F; femur, Fib; fibula, H; humerus, R; radius, T; tibia, U; ulna .

| **Distance from longitudinal origin (um)** | **E17.5** | | **E18.5** | | **P14** | | **P16** | | **P32** | | **P40** | |
| --- | --- | --- | --- | --- | --- | --- | --- | --- | --- | --- | --- | --- |
|  | **mean** | **std** | **mean** | **std** | **mean** | **std** | **mean** | **std** | **mean** | **std** | **mean** | **std** |
| **F-D** | 755.54 | 40.24 | 1054.53 | 31.58 | 3924.00 | 50.91 | 4356.00 | 101.82 | 6030.00 | 25.46 | 6822.00 | 25.46 |
| **Fib-D** | 786.72 | 50.33 | 1084.11 | 36.27 | 3960.00 | 36.00 | 4512.00 | 109.98 | 5580.00 | 101.82 | 5580.00 | 101.82 |
| **Fib-P** | 788.15 | 76.32 | 1119.80 | 56.73 | 4116.00 | 20.78 | 4380.00 | 166.28 | 6084.00 | 50.91 | 6714.00 | 25.46 |
| **H-P** | 855.79 | 78.80 | 1173.47 | 28.03 | 3393.00 | 90.00 | 3681.00 | 79.82 | 4959.00 | 45.30 | 5472.00 | 105.98 |
| **R-D** | 882.56 | 92.68 | 1270.74 | 42.66 | 4779.00 | 139.04 | 5328.00 | 124.71 | 6660.00 | 95.25 | 7080.00 | 145.49 |
| **T-D** | 722.82 | 12.19 | 1029.44 | 70.56 | 3708.00 | 92.95 | 4005.00 | 90.00 | 5040.00 | 50.91 | 5148.00 | 0.00 |
| **T-P** | 890.59 | 37.04 | 1242.19 | 19.55 | 4518.00 | 139.43 | 5013.00 | 156.58 | 7146.00 | 127.28 | 7830.00 | 229.10 |
| **U-D** | 887.28 | 84.04 | 1223.83 | 40.77 | 5148.00 | 224.82 | 5676.00 | 177.58 | 7605.00 | 118.95 | 8055.00 | 129.38 |
|  |  |  |  |  |  |  |  |  |  |  |  |  |
| **Equivalent Radius (um)** | **E17.5** | | **E18.5** | | **P14** | | **P16** | | **P32** | | **P40** | |
|  | **mean** | **std** | **mean** | **std** | **mean** | **std** | **mean** | **std** | **mean** | **std** | **mean** | **std** |
| **F-D** | 279.20 | 9.91 | 331.90 | 4.89 | 827.50 | 22.38 | 859.77 | 30.54 | 1038.39 | 13.48 | 1035.63 | 9.01 |
| **Fib-D** | 116.33 | 8.53 | 126.58 | 2.78 | 272.41 | 16.78 | 252.04 | 2.46 | 247.07 | 4.72 | 238.59 | 2.45 |
| **Fib-P** | 125.17 | 1.76 | 143.79 | 6.15 | 312.20 | 15.52 | 300.53 | 5.87 | 436.13 | 18.39 | 414.00 | 4.58 |
| **H-P** | 300.57 | 10.33 | 357.05 | 8.30 | 813.67 | 18.74 | 829.80 | 8.15 | 942.19 | 13.88 | 933.65 | 22.82 |
| **R-D** | 170.56 | 6.80 | 206.55 | 6.78 | 470.33 | 17.03 | 473.17 | 3.92 | 484.29 | 16.96 | 446.07 | 14.36 |
| **T-D** | 199.66 | 3.94 | 238.99 | 3.48 | 543.17 | 12.98 | 548.26 | 7.94 | 625.07 | 27.07 | 636.28 | 22.46 |
| **T-P** | 258.71 | 6.54 | 314.58 | 7.13 | 781.54 | 18.47 | 834.36 | 16.01 | 1096.62 | 17.96 | 1123.73 | 28.55 |
| **U-D** | 151.62 | 5.10 | 181.86 | 5.16 | 312.23 | 14.65 | 302.18 | 14.19 | 331.93 | 15.30 | 304.65 | 16.77 |
